# Diagnosis of mixed infection and a primary immunodeficiency disease using next-generation sequencing: a case report

**DOI:** 10.3389/fcimb.2023.1179090

**Published:** 2023-08-22

**Authors:** Xiaolei Zhang, Yixue Wang, Daly Pen, Jing Liu, Qinhua Zhou, Yao Wang, Huaqing Zhong, Tingyan Liu, Weiming Chen, Bingbing Wu, Yang Zhou, Chuanqing Wang, Xiangyu Li, Fangyou Yu, Xiaochuan Wang, Guoping Lu, Gangfeng Yan

**Affiliations:** ^1^ Department of Pediatric Intensive Care Unit, Children’s Hospital of Fudan University, Shanghai, China; ^2^ Department of Pediatric Intensive Care Unit, Angkor Hospital for Children, Siem Reap, Cambodia; ^3^ Department of Allergy and Clinical Immunology, Children’s Hospital of Fudan University, Shanghai, China; ^4^ Department of Pediatric Institute, Children’s Hospital of Fudan University, Shanghai, China; ^5^ Shanghai Key Laboratory of Birth Defects, Children’s Hospital of Fudan University, Shanghai, China; ^6^ National Health Commision Key Laboratory of Neonatal Diseases, Children’s Hospital of Fudan University, Shanghai, China; ^7^ Clinical Research Department, BGI PathoGenesis Pharmaceutical Technology Co., Ltd, BGI-Shenzhen, Shenzhen, China; ^8^ Clinical Microbiology Laboratory, Department of Nosocomial Infection Control, Children’s Hospital of Fudan University, Shanghai, China; ^9^ Department of Laboratory Medicine, Huashan Hospital North, Fudan University, Shanghai, China; ^10^ Shanghai Key Laboratory of Tuberculosis, Shanghai Pulmonary Hospital, Tongji University School of Medicine, Shanghai, China

**Keywords:** major histocompatibility complex class II, immunodeficiency, mycobacterium abscessus, next-generation sequencing, whole exome sequencing

## Abstract

Major Histocompatibility Complex Class II (MHC II) deficiency is a rare primary immunodeficiency disorder (PID) with autosomal recessive inheritance pattern. The outcome is almost fatal owing to delayed diagnosis and lacking of effective therapy. Therefore, prompt diagnosis, timely and effective treatment are critical. Here, we report a 117-day-old boy with diarrhea, cough, cyanosis and tachypnea who was failed to be cured by empiric antimicrobial therapy initially and progressed to severe pneumonia and respiratory failure. The patient was admitted to the pediatric intensive care unit (PICU) immediately and underwent a series of tests. Blood examination revealed elevated levels of inflammatory markers and cytomegalovirus DNA. Imaging findings showed signs of severe infection of lungs. Finally, the diagnosis was obtained mainly through next-generation sequencing (NGS). We found out what pathogenic microorganism he was infected via repeated conventional detection methods and metagenomic next-generation sequencing (mNGS) of sputum and bronchoalveolar lavage fluid (BALF). And his whole exome sequencing (WES) examination suggested that CIITA gene was heterozygous mutation, a kind of MHC II deficiency diseases. After aggressive respiratory support and repeated adjustment of antimicrobial regimens, the patient was weaned from ventilator on the 56th day of admission and transferred to the immunology ward on the 60th day. The patient was successful discharged after hospitalizing for 91 days, taking antimicrobials orally to prevent infections post-discharge and waiting for stem cell transplantation. This case highlights the potential importance of NGS in providing better diagnostic testing for unexplained infection and illness. Furthermore, pathogens would be identified more accurately if conventional detection techniques were combined with mNGS.

## Introduction

Major Histocompatibility Complex Class II (MHC II) deficiency, a primary immunodeficiency disease (PID), is extremely difficult to be diagnosed in children in their early lives. The clinical manifestations of PIDs are diverse, and infection is one of the most common manifestations (Bousfiha et al., 2020). Patients with MHC II deficiency typically manifest as severe respiratory and gastrointestinal infections. Such patients are susceptible to various pathogens, including viruses, bacteria, fungi and parasite, resulting in high infant mortality. Therefore, it is very important to identify the causative pathogens timely and the etiology early for managing the disease effectively. So far, conventional methods are limited in pathogen identification in immunocompromised patients (Bousfiha et al., 2020; Tangye et al., 2020) and metagenomic next-generation sequencing (mNGS) has been widely used to identify pathogens since 2005 (Voelkerding et al., 2015). Besides, NGS has boosted the discovery of novel genetic etiologies of known and novel PID phenotypes since 2010. NGS based gene panel sequencing, whole exome sequencing (WES), is an ideal method in this field, for research or diagnostic goals (Meyts et al., 2016). In this report, we described the potential contribution of NGS in timely diagnosing multiple pathogens and etiology, providing evidence to manage and diagnose MHC II deficiency patient effectively, resulting in favorable clinical outcomes.

## Case report

**Figure 1:**
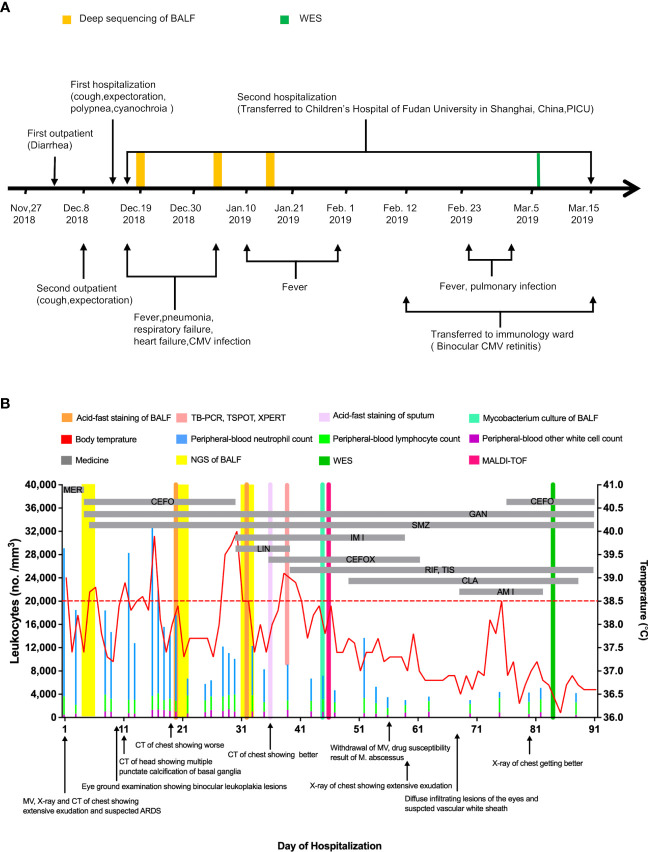
Clinical Course of the 117-day-old Patient with mixed infection and primary disease. **(A)** shows a timeline beginning with the patient’s first vsit to a regional hospital in November 2018 and ending after his recovery in March 2019. Major events during the course of the patient’s illness are indicated by arrows. **(B)** shows laboratory values obtained and pertinent medications administered during the patient’s second hospitalization. The graph shows the body-temperature curve (red line) and peripheral-blood neutrophil counts (blue bars), lymphocyte counts (green bars) and other white cell count (purple bars). The horizontal thick gray lines show the medications administered. (MER denotes meropenem, CEFO denotes cefoperazone sulbactam, SMZ denotes cotrimoxazole, GAN denotes ganciclovir, IMI denotes imipenem cilastatin sodium, LIN denotes linezolid, CEFOX denotes cefoxitin, RIF denotes rifampin, TIS denotes tisoniazid, matrix-assisted laser desorption ionization-time of flight denotes MALDI-TOF.

On December 14^th^, 2018, a 117-day-old infant was admitted to the emergency department of the Children’s Hospital of Fudan University in Shanghai, China, after continuous diarrhea for 2 weeks, cough for 1 week, and shortness of breath for 1 day (**Figure 1A**). Fourteen days previously, on December 1^st^, 2018, he visited the outpatients’ department of a regional hospital for diarrhea, and his symptoms were relieved after probiotic treatment (**Figure 1A**). On December 8^th^, 2018, he visited the same regional hospital again for cough and expectoration and was diagnosed with pneumonia and treated with cefaclor (**Figure 1A**). Six days later, he was hospitalized with progressively severe pneumonia and respiratory failure (**Figure 1A**). Mask oxygen inhalation was given, and ceftriaxone and azithromycin were administered.

**Figure 2:**
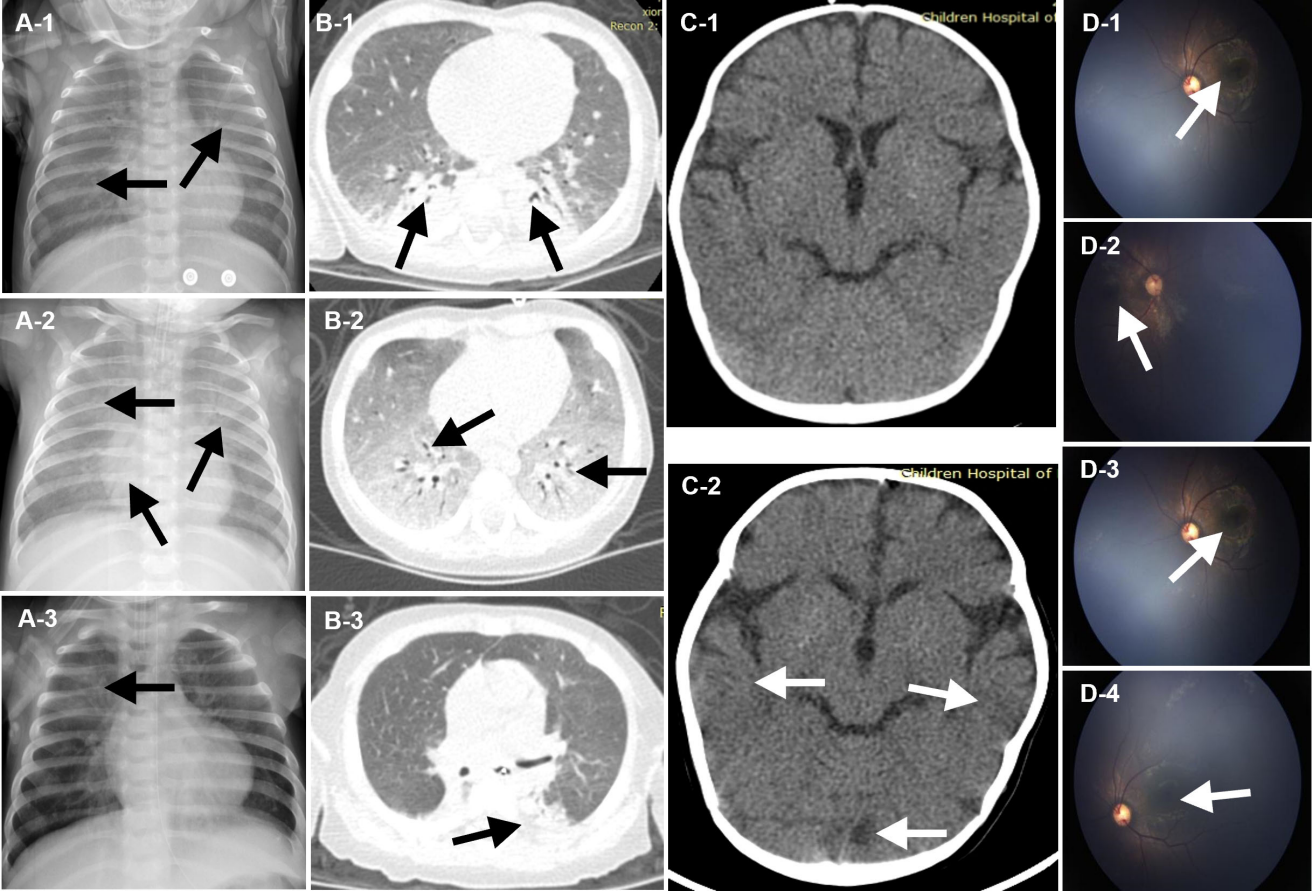
Chest Imaging, Neuroradiologic CT and Fundus perforator. The images shown in **(A-1)**, **(B-1)**, and **(C-1)** were acquired on admission to the emergency department of Children’s Hospital of Fudan University. Extensive exudation and parenchymal lesion of the chest was revealed (**A-1**, **B-1**, black arrows). And the image of the head was normal (**C-1**, white arrows). The image shown in **(A-2)**, **(B-2)**, and **(C-2)**, **(D-1)** and **(D-2)** were acquired on day 14, after 10-day anti-microbial therapy. Progression of parenchymal lesion and interstitial infiltrates of the chest was revealed (**A-2**, **B-2**, black arrows), while multiple punctate calcifications of basal ganglia of the head was revealed (**C-2**, white arrows). Fundus examination suggested binocular leukoplakia lesions (**D-1**, **D-2**, white arrows). The image shown in **(A-3)** and **(F)** were acquired on day 36, after targeted anti-microbial for multiple pathogens. Lesions in lungs was nearly resolved (**A-3**, **B-3**, black arrows). The image shown in **(D-3)**, **(D-4)** were acquired on day 72. Diffuse infiltrating lesions in his eyes and vascular white sheath were revealed (**D-3**, **(D-4)**, white arrows).

After brief hospitalization in a regional hospital, the patient’s progressively worsening condition so that he was transferred to the emergency department of our hospital for further evaluation and treatment (**Figure 1A**). On admission, his clinical symptoms are mainly dyspnea and cyanosis, respiratory distress with crackles in both lungs on auscultation. He was immediately admitted to the pediatric intensive care unit (PICU). High-flow nasal cannula oxygen therapy was initiated. About 17 hours later, there was no improvement of respiratory distress, so the patient was intubated and received mechanical ventilation (MV) (**Figure 1B**). The initial peripheral blood leukocyte count was 29100/mm3, with 86.3% neutrophils and 10.9% lymphocytes (**Figure 1B**), C-reactive protein (CRP) was 12 mg/L, and procalcitonin (PCT) was 0.35 ng/ml. Chest X-ray and computed tomography (CT) imaging revealed extensive exudation (**Figures 2A-1, B-1**). The CT of head was normal (**Figure 2C-1**). Given the high suspicion of a severe bacterial infection, meropenem (MER) was empirically administered.

From the 1^st^ day to the 5^th^ day, his pulmonary signs did not improve and he had an intermittent fever, reaching up to 39.0°C (**Figure 1B**). Acid-fast staining of the sputum and bronchoalveolar lavage fluid (BALF) were negative on the 4^th^ day. Bacterial cultures of sputum and BALF were P. aeruginosa, that was sensitivity to third-generation cephalosporins. Serum viral CMV-DNA was 3.2×10^6^ copies/L on the 5^th^ day. mNGS of BALF showed several potential pathogens including P. aeruginosa, P. jirovecii, and CMV on the 6^th^ day (**Supplementary Figure 1**, **Supplementary Table 1**). The patient was treated with cefoperazone sulbactam (CEFO), co-trimoxazole (SMZ), and ganciclovir (GAN). On the 10^th^ Day, a repeat X-ray and chest CT scan showed the progression of parenchymal lesions and interstitial infiltrates (**Figures 2A-2, B-2**), and the head CT scan suggested multiple punctate calcifications of the basal ganglia (**Figure 2C–2**). Fundus examination suggested binocular leukoplakia lesions (**Figures 2D-1, D-2**). Therefore, CEFO, SMZ, GAN treatments were continued.

On the 18^th^ day, the patient’s temperature dropped to 37.4°C. On the 22^nd^ day, a repeated mNGS of BALF revealed that CMV and P. jirovecii sequences decreased significantly and a new pathogen, M. abscessus, was found (**Supplementary Figure 1**, **Supplementary Table 1**). The second acid-fast staining analysis of BALF was positive. However, the T-Cell spot test (T-SPOT), the purified protein derivative test (PPD), the M. tuberculosis polymerase-chain-reaction (TB-PCR) assay, and BALF culture results were all negative (**Figure 1B**). The patient’s body temperature returned to normal and his pulmonary infiltrates improved after his admission. Based on these above, it is considered that the evidence of M. abscessus infection is insufficient.

**Figure 3:**
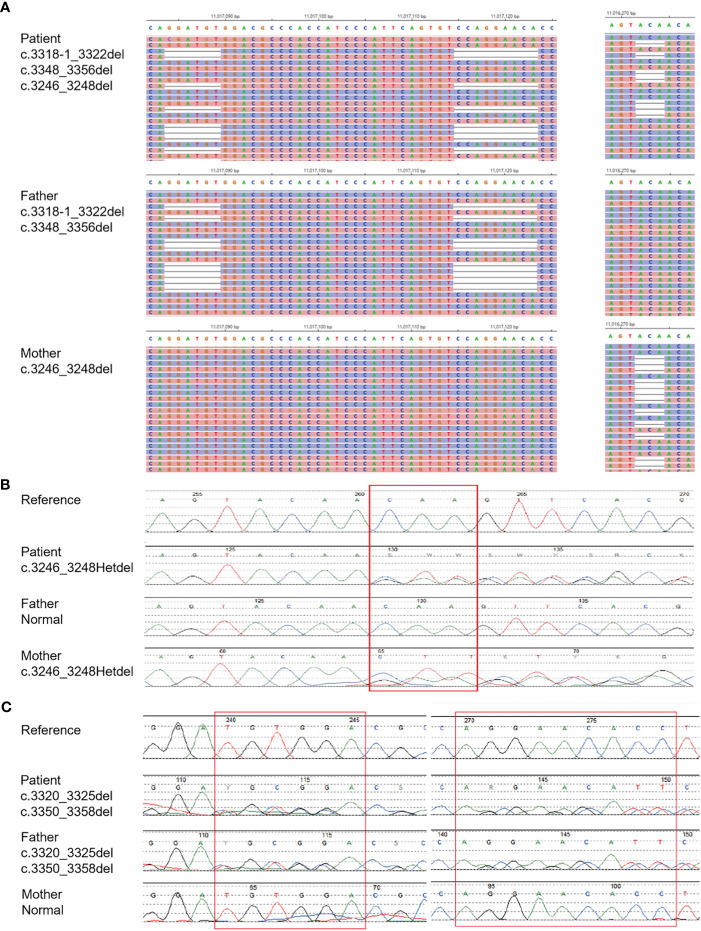
Diagnosis of MHC II deficiency by Means of Unbiased NGS. **(A)** shows the result of whole exome sequencing. **(B, C)** shows the result of generation sequencing.

On the 28^th^ day, the patient developed a new onset of fever, and his body temperature rose to 40°C (**Figure 1B**). The third acid-fast staining analysis on sputum and BALF remained positive. On the 34^th^ day, the third mNGS on BALF suggested an increase sequences in M. abscessus (**Supplementary Figure 1**, **Supplementary Table 1**). Although the Xpert test, T-SPOT, and TB-PCR remained negative, M. abscessus infection could not be ruled out. CEFO was discontinued, and imipenem cilastatin sodium (IMI), linezolid (LIN) and cefoxitin (CEFOX) were administered. There was no improvement in clinical about 7 days later, and LIN was replaced with rifampin (RIF) and tisoniazid (TIS). In the following days, his lung lesions resolved and his temperature dropped close to normal (**Figures 2A-3, B-3**). On the 45^th^ day, M. abscessus was obtained from the BALF by matrix-assisted laser desorption ionization-time of flight (MALDI-TOF) after prolonged incubation. Finally, clarithromycin (CLA), CEFOX and IMI were administered in accordance with the drug susceptibility tests.

After adjusting the treatment, patient’s condition gradually improved. On the 56^th^ day, he was eventually weaned from ventilator and transferred to the immunology ward on the 60^th^ day (**Figures 1A, B**). In the immunology ward, amikacin and CEFO were used against P. aeruginosa and Acinetobacter baumannii. Fundus reexamination showed diffuse infiltrating eye lesions and a vascular white sheath (**Figures 2D-3, D-4**). On the 75^th^ day, the CMV-DNA in the humor aqueous decreased from 4.1×10^6^ copies/ml to 1.3×10^4^ copies/ml after an anterior intraocular injection with GAN. Reexamination of the serum CMV-DNA decrease to 4.14 × 10^4^ copies/L.

The patient was infected with a variety of pathogens in his early life. Repeated peripheral blood examinations suggested a low proportion of lymphocytes (<10%). Immunoglobulin and serum complements were nearly normal (**Table 1**). Lymphocyte subsets revealed an inverted cluster of differentiation CD4+/CD8+ ratio and significant decreases in NK cells and CD4+ T cells (**Table 1**). Immunodeficiency diseases needed to be considered, and during hospitalization, the WES of his family was performed after obtaining written consent. After 3 months, the results showed MHC Class II deficiency, which was caused by a heterozygous mutation in the class II major histocompatibility complex transactivator (CIITA) gene (**Figure 3A**).

**Table 1:** Assessment of immune function during hospitalization.

Immunologic index	Dec. 17^th^, 2018	Dec. 26^th^, 2018	Jan.11^th^, 2019	Jan.23^th^, 2019
Lymphocytes subgroup				
CD3 (%)	47.71	58.97	74.63	73.8
CD3 counts (/ml)	616.1	1136.6	1213.1	789.7
CD4 (%)	6.38	10.34	12.12	16.18
CD4 counts (/ml)	82.36	199.34	196.89	173.15
CD8 (%)	40.49	49.97	61.39	55.97
CD8 counts (/ml)	522.98	963.01	997.44	598.97
CD4/CD8	0.16	0.21	0.2	0.29
CD19 (%)	49.26	36.1	20.46	16.63
CD19 counts (/ml)	636.22	695.67	332.48	177.96
CD16 CD56 (%)	0.96	3.96	4.21	7.23
CD16 CD56 counts (/ml)	12.35	76.27	68.44	77.37
CD45 counts (/ml)	1291.493	1927.258	1624.777	1070.138
Immunoglobulin				
IgG (g/L)	16.40	12.30	17.00	^a^
IgA (g/L)	0.37	0.26	0.39	^a^
IgM (g/L)	2.53	1.88	0.63	^a^
Complements				
C3 (g/L)	0.36	0.67	^a^	^a^
C4 (g/L)	0.08	0.16	^a^	^a^
CH50 (U/ml)	14	32	^a^	^a^
IgE (KU/L)	38.19	28.62	^a^	^a^

a equals no test results at the corresponding time point.

The patient was discharged on the 91^st^ day after a 12-week course of GAN and SMZ, a 7-week course of RIF and TIS, a 6-week course of CLA, a 5-week course of IMI, and a 4-week course of CEFOX, with a status close to his premorbid condition. He still had to take antimicrobials orally for prophylaxis against opportunistic infections post-discharge. Active follow ups are required, and transplant therapy is anticipated.

## Method

Analysis of the patient’s clinical samples for the identification of potential pathogens and etiology was approved by the institutional review board at the Children’s Hospital of Fudan University in Shanghai, China (No.2020494). Samples were processed in a medical laboratory. BALF samples (0.5-3.0 mL) were heat inactivated (65°C, 30 min) before mixing with 0.5 mL glass beads (0.5 mm) in a sterile 1.5-mL microcentrifuge tube. The mixture was attached to a horizontal platform on a vortex mixer and agitated vigorously (2800-3200 rpm, 20 min). Samples (0.3 mL) were added to a new 1.5-mL microcentrifuge tube and DNA was extracted using the TIANamp Micro DNA Kit (DP316, TIANGEN BIOTECH) in accordance with the manufacturer’s recommendations.

DNA libraries were constructed with MGIEasy DNA Library Prep Kit by fragmentation, end-repair, adaptor ligation, and PCR amplification under the following conditions: 98°C for 2 minutes, followed by 12 cycles of 98°C for 15 s, 56°C for 15 s, and 72°C for 30 s, with a final extension at 72°Cfor 5 minutes. The Agilent 2100 Bioanalyzer system (Agilent, Santa Clara, CA, USA) and the Qubit dsDNA HS Assay Kit (Thermo Fisher Scientific Inc., Waltham, MA, USA) was used to control the DNA library fragment sizes and concentrations (200-300 bp, >2 ng/μL). The libraries were sequenced on the BGISEQ-50 platform (Jeon et al., 2014).

High-quality sequencing data were generated by removing low-quality and short (< 35 bp long) reads, and the human host sequences mapped to the human reference genome (hg19) were computationally subtracted using Burrows-Wheeler Alignment (Li and Durbin, 2009). After removing low-complexity reads, the remaining sequences were simultaneously aligned in four microbial genome databases consisting of the genomes or scaffolds from 4945 viruses, 6350 bacteria (including 174 Mycobacterium spp. and 137 Mycoplasma/Chlamydia), 1064 fungi, and 234 parasites downloaded from the National Center for Biotechnology Information (NCBI; ftp://ftp.ncbi.nlm.nih.gov/genomes/).

## Results

### Rapid identification of mixed pathogen sequences in BALF

mNGS runs comprised 10 individual samples and a blank control. At least 20 million raw reads were produced from a single BALF specimen. The sequence reads from specific microbes were reported as strictly mapped read numbers per 20 million of the total sequences (SDSMRN). Multiple pathogens were detected in the first analysis. P. jirovecii was the dominant fungus (99.92% relative abundance; genome coverage, 25.11%; depth 1.18 X) (**Supplementary Figure 1**, **Supplementary Table 1**). The dominant virus was CMV (89.94% relative abundance; genome coverage, 54.07%; depth 1.8 X). P. aeruginosa was also detected (9.6% relative abundance; genome coverage, 0.1245%; depth 1 X) (**Supplementary Figure 1**, **Supplementary Table 1**). In the second detection post-antimicrobial treatment, the SDSMRN for P. jirovecii and CMV decreased from 23,211 to 769 and 2,141 to 79, respectively (**Supplementary Figure 1**, **Supplementary Table 1**). However, the SDSMRN for P. aeruginosa rose from 65 to 592, accompanied by an increased relative abundance from 9.6% to 27.57% (**Supplementary Figure 1**, **Supplementary Table 1**). A new bacterium, M. abscessus, was identified as a possible pathogen (SDSMRN, 95; 6.05% relative abundance; genome coverage, 0.2395%; depth 1X) (**Supplementary Figure 1**, **Supplementary Table 1**). In the third detection, the SDSMRN for M. abscessus increased to 321, with an increased genome coverage to 1.02% (**Supplementary Figure 1**, **Supplementary Table 1**). The presence of P. jirovecii was validated by PCR and Sanger sequencing. CMV was confirmed by testing serum CMV-DNA. P. aeruginosa was confirmed by bacterial culture on both sputum and BALF. M. abscessus was confirmed by bacterial culture on BALF and by MALDI-TOF.

### Accurate identification of immunodeficiency disease using blood plasma

WES analysis on the blood plasma detected compound heterozygous mutations (c.3318_c.3322delGGATAT and c.3348_3356delCCAGGAACA, paternal; and c.3246_3248delCAA, maternal) in the CIITA gene on chromosome 16 (**Figure 3A**). These mutations were validated by NGS (**Figures 3B, C**).

## Discussion

In this report, we describe the main process of diagnosis and treatment of a patient, who began with mixed infection of respiratory and digestive tract initially and was eventually diagnosed with MHC II deficiency. The NGS, a form of gene technology, that runs through the whole process in this case and plays a key role, was found to be a promising method for finding pathogens and pathogenic genes.

MHC II plays an important role in the development and regulation of immune system. MHC II deficiency results in a decreased number and impaired antigen presentation of the CD4^+^ T cell. The peripheral blood shows a significant decrease in lymphocyte. The sub-lymphocyte population shows significantly decreased CD4^+^ T cell and a inverted CD4^+^/CD8^+^ ratio. MHC II deficiency typically presents with respiratory tract and gastrointestinal infections, and its atypical manifestations includes septicaemia, neurological symptoms, rarely autoimmune diseases, infection after live vaccination and so on (Dimitrova et al., 2014; Alyasin et al., 2015; Matzaraki et al., 2017). The definitive diagnosis is made by genetic analysis, usually based on the absence or limited expression of HLA-DR or DP on B cells or monocytes using flow-cytometry (Farrokhi et al., 2018). MHC II deficiency was first reported in 1979, and to date, about 200 cases with MHC II deficiency have been reported worldwide (Hanna and Etzioni, 2014). However, only a few cases have been reported so far in China. There are two types of regulatory genes that regulate the expression of MHC II. One is CIITA, the other one is the RFX complex (Jahnavi et al., 2018). CIITA was the first described gene that caused MHC II deficiency disease. CIITA deficiency in European countries has a high prevalence (~11%), and approximately 12 different mutations have been reported (Mannhalter et al., 1991; Steimle et al., 1993; Mach et al., 1996; Bontron et al., 1997; Fondaneche et al., 1998; Quan et al., 1999; Peijnenburg et al., 2000; Wiszniewski et al., 2001; Dziembowska et al., 2002; Schmetterer et al., 2010; Ouederni et al., 2011; Dimitrova et al., 2014; Farrokhi et al., 2018) (**Supplementary Table 2**). For this patient, his first sub-lymphocyte population showed significantly decreased CD4^+^ T cell, but the reexamined CD4^+^ T cell count increased to nearly normal. And there was little change in serum immunoglobulin. Additionally, HLA-DR expression did not decrease (**Supplementary Figure 2**). The genetic mutations site found in this patient have not been reported in the scientific literature yet. All the findings may be related to the specific site of gene CIITA, which are located at the end of the protein encoded by it, which may only partially affect the protein’s function.

In this case, the patient initially developed with respiratory and gastrointestinal symptoms in his 117 days. Considering that he may be immunocompromised, early rapid pathogen identification is the key to treatment. However, identifying all pathogens in the meantime is a big challenge. Currently, mNGS, a popular and well-established method for pathogen detection has been commonly used. Studies showed that mNGS can identify all microorganisms, including rare or non-cultivable ones, in a single-step with samples from immunocompromised patients under antimicrobial therapy (Parize et al., 2017). Different pathogens can be simultaneously detected in a single specimen, such as blood, sputum, nasopharyngeal swabs, BALF, cerebrospinal fluid, and tissues, among others (Thorburn et al., 2015; Guan et al., 2016; Long et al., 2016; Ai et al., 2018). After admission, the conventional laboratory examination showed our patient was infected with P. aeruginosa and CMV. At the same time, the mNGS of BALF showed not only the pathogens mentioned above but also P. jirovecii. That shows that mNGS might have an advantage over conventional microbiological methods in detecting mixed infections. After adjusting the antimicrobial regimen, the patient’s condition was improved. The second culture of BALF turned out to be negative, and the sequence of CMV and P. jirovecii in the second mNGS of BALF decreased. But the second acid-fast staining of BALF was positive and a new pathogen, M. abscessus, emerged in the second mNGS of BALF at a low relative abundance. However, combining his clinical condition with negative results of a series tests for Mycobacteria, M. abscessus were not considered. Shortly afterwards, his condition relapsed. Although the third culture of BALF was still negative, the acid-fast staining was positive and the mNGS showed a increased sequence of M. abscessus. With these results, we were alerted to a possible infection of M. abscessus. Therefore, we prolonged the time of BALF culture, which later confirmed M. abscessus as the causative microorganism.

mNGS is a direct, non-specific technique, which can detect all nucleic acid fragments without prior selection of the reference range to avoid missing pathogen, but false negatives still occurred (Zhang et al., 2019). When we find pathogen though mNGS, it is important to confirm whether the pathogen are pathogenic, especially for patients with negative culture results or poor responses to empirical antibiotic therapy. In this case, M. abscessus was detected as early as the second mNGS of BALF, which showed an advantage than conventional methods. If we correctly interpreted the result of the second mNGS of BALF, perhaps it would have greatly shortened the length of hospitalization. The turnaround time of mNGS was 24-48 hours. With the localization and automation of the assay and the shortening of the sequencing time, the turnaround time was expected to be further reduced to less than 24h. mNGS assay required technicians to master special skills like nucleic acid extraction, library construction, sequencing and bioinformatics analysis. Standardized processes and quality control of mNGS were key points, which can directly affect the accuracy of mNGS results and the feasibility of comparing the results from different assays. Currently, there is no recognized standard of mNGS interpretation, and it is not clear how to interpret the sequence coverage and number better of a pathogen with clinician’s opinion. Retrieval of mNGS raw data and validation with other clinical assays is the current preferred solution. In the future, the improvements in bacterial resistance gene sequencing, host genomics, mNGS sequencing cost and their therapeutic-related interpretation shall enhance the clinical value of mNGS in infectious diseases. In this report, we identified the pathogen primarily by combining bacterial culture and mNGS. Although conventional microbiological methods are time-consuming and complicated, have high false-negative rates, they still play an important role (Mwaigwisya et al., 2015). We believe that pathogens would be identified more accurately if we combined conventional detection techniques with mNGS.

## Conclusions

In summary, NGS is not only used to identify the genetic deficiency, but also to detect pathogens earlier, more rapidly and comprehensively, which highlights the potential of NGS in pathogen detection and rapid etiological diagnosis, helping physicians on diagnosis and medical countermeasures to save lives. Furthermore, pathogens would be identified more accurately if conventional detection techniques were combined with mNGS.

## Data availability statement

The data presented in the study are deposited in the GenBank repository, accession numberPRJNA1002760.

## Ethics statement

Written informed consent was obtained from the minor(s)’ legal guardian/next of kin for the publication of any potentially identifiable images or data included in this article.

Written informed consent was obtained from the participant/patient(s) for the publication of this case report

## Author contributions

XZ, YXW and DP contributed equally to this work as co-first authors. XZ, YXW and DP wrote the first and revised the manuscript. GL and GY conceptualized and designed the study, and took responsibility for the integrity of the whole work. They contributed equally to this work as co-corresponding authors. The others performed the collection, analysis, interpretation of data. All authors contributed to the article and approved the submitted version.

## Supplementary material

Supplementary Figure 1 Diagnosis of mixed Infection by Means of Unbiased mNGS. The distribution of pathogen sequences identified in the patient’s BALF included P. jiroveci and M. Abscess, which are generally considered to be pathogenic groups, and CMV and P. aeruginosa, which are generally considered to be colonization bacteria groups.

Supplementary Figure 2 The expression of HLA-DRα in human.
