# Supplementary figures and images for: Diagnosis of mixed infection and a primary immunodeficiency disease using next-generation sequencing: a case report
Source: Front Cell Infect Microbiol. 2023 Aug 22;13:1179090. doi: 10.3389/fcimb.2023.1179090 (PMC10477990; doi:10.3389/fcimb.2023.1179090)

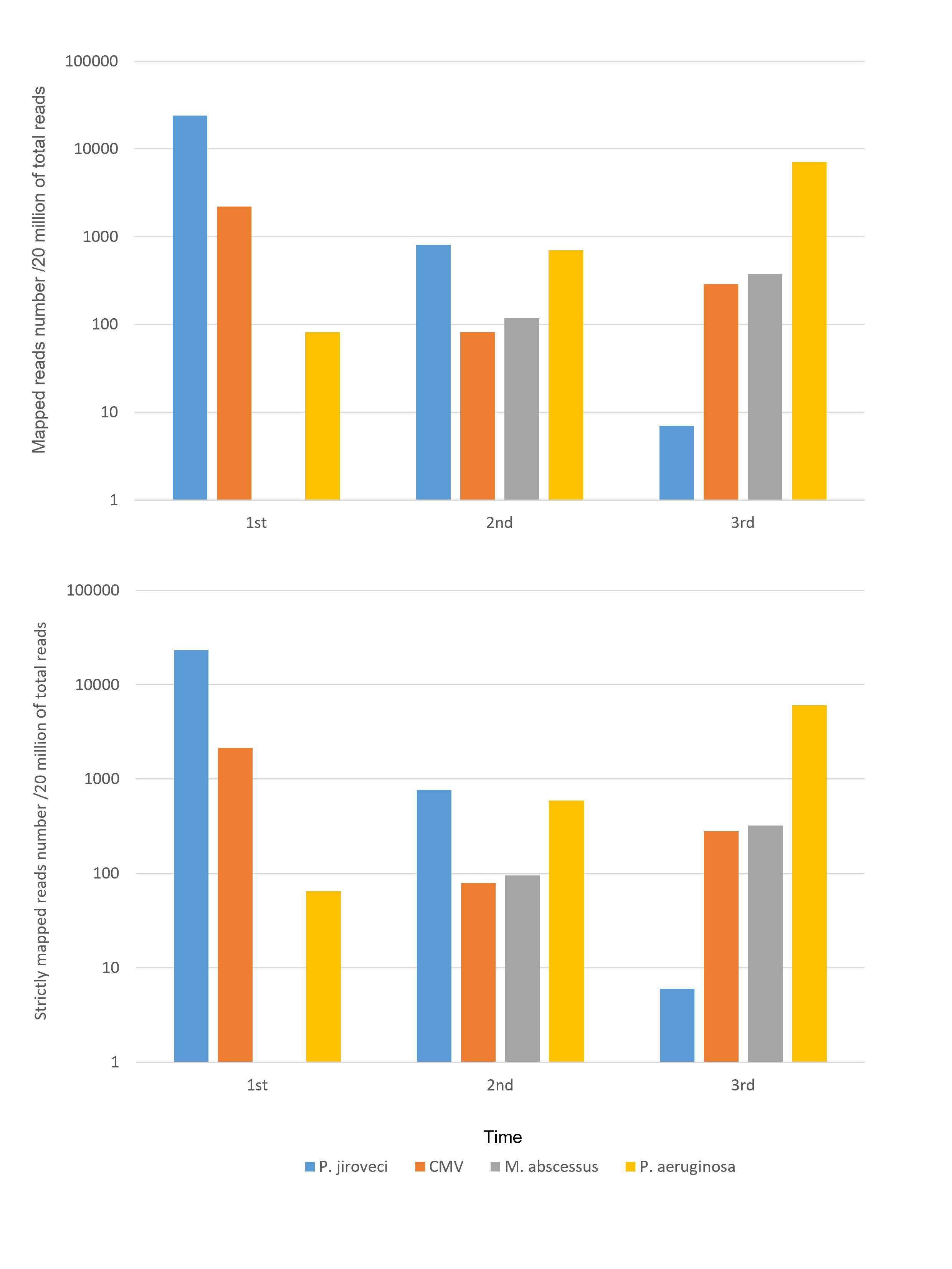

Supplement: Supplementary Figure 1 — Diagnosis of mixed Infection by Means of Unbiased mNGS. The distribution of pathogen sequences identified in the patient’s BALF included P. jiroveci and M. Abscess, which are generally considered to be pathogenic groups, and CMV and P. aeruginosa, which are generally considered to be colonization bacteria groups. [file Image_1.tif]

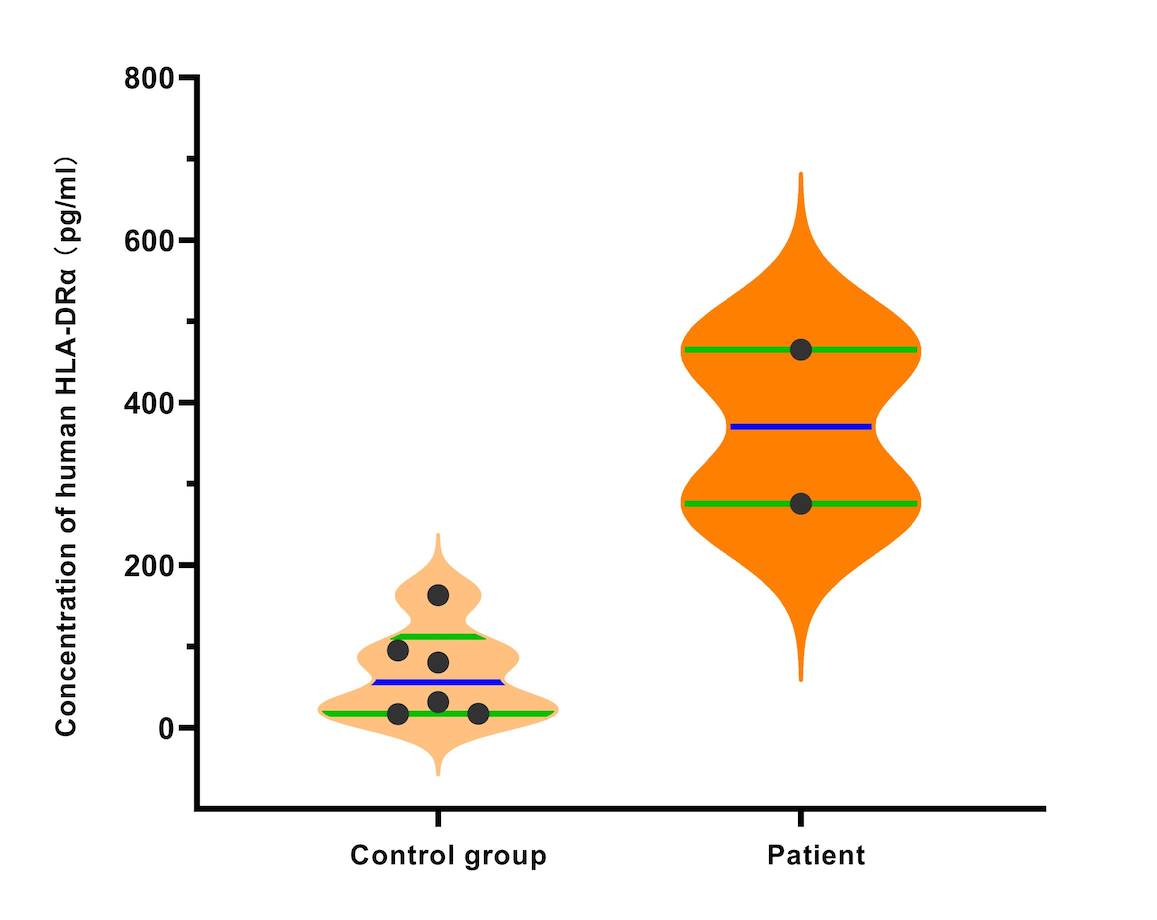

Supplement: Supplementary Figure 2 — The expression of HLA-DRα in human. [file Image_2.tiff]
