# Supplementary material for: Diagnosis of mixed infection and a primary immunodeficiency disease using next-generation sequencing: a case report
Source: Front Cell Infect Microbiol. 2023 Aug 22;13:1179090. doi: 10.3389/fcimb.2023.1179090 (PMC10477990; doi:10.3389/fcimb.2023.1179090)
Supplement: Supplementary file 3 [file Table_1.docx]

| Supplementary Table 1. The basic situation of pathogens in triple NGS-based detections | | | | |
| --- | --- | --- | --- | --- |
| Pathogens | Time | Dec. 18^th^, 2018 | Jan. 3^rd^, 2019 | Jan. 14^th^, 2019 |
|  |  | 1st | 2nd | 3rd |
| P. jiroveci | SDMRN^a^ | 23943 | 803 | 7 |
|  | SDSMRN ^b^ | 23211 | 769 | 6 |
|  | genome coverage (%) | 25.11 | 1.01 | 0.0113 |
|  | depth | 1.18 | 1.01 | 1 |
|  | relative abundance in the Kingdom(%) | 99.92 | 99.53 | 85.05 |
| CMV | SDMRN ^a^ | 2209 | 82 | 289 |
|  | SDSMRN ^b^ | 2141 | 79 | 279 |
|  | genome coverage (%) | 54.07 | 3.61 | 15.32 |
|  | depth | 1.8 | 1.02 | 1.13 |
|  | relative abundance in the Kingdom(%) | 89.94 | 86.36 | 82.94 |
| M. abscessus | SDMRN ^a^ | 0 | 117 | 379 |
|  | SDSMRN ^b^ | 0 | 95 | 321 |
|  | genome coverage (%) | 0 | 0.2395 | 1.02 |
|  | depth | 0 | 1 | 1 |
|  | relative abundance in the Kingdom(%) | 0 | 6.05 | 4.39 |
| P. aeruginosa | SDMRN ^a^ | 82 | 698 | 7057 |
|  | SDSMRN ^b^ | 65 | 592 | 6021 |
|  | genome coverage (%) | 0.1245 | 1.08 | 13.19 |
|  | depth | 1 | 1.01 | 1.11 |
|  | relative abundance in the Kingdom(%) | 9.6 | 27.57 | 62.22 |

^a^ mapped reads number /20 million of total reads, ^b^ strictly mapped reads number /20 million of total reads.
