# Supplementary material for: Diagnosis of mixed infection and a primary immunodeficiency disease using next-generation sequencing: a case report
Source: Front Cell Infect Microbiol. 2023 Aug 22;13:1179090. doi: 10.3389/fcimb.2023.1179090 (PMC10477990; doi:10.3389/fcimb.2023.1179090)
Supplement: Supplementary file 4 [file Table_2.docx]

| Supplementary Table 2. Clinical data. | | | | | | |
| --- | --- | --- | --- | --- | --- | --- |
| Patient | Region | Clinical features | Mutation | Treatment | Outcome | Study/ Reference |
| 1 | Austrian | NM ^a^ | Allele2: 84bp del3349-3432/ p.D1078E | NM ^a^ | NM ^a^ | Mannhalter et al. (1991) (11) |
|  |  |  | Allele1:c.G1256T (p. E381X) |  |  | Bontron et al. (1997) (12) |
| 2 | NM ^a^ | NM ^a^ | Homozygous c. 72bp del2932-3003 | NM ^a^ | NM ^a^ | Steimle et al. (1993) (13) |
| 3 | Pakistani | Recurrent infections, severe diarrhoea and failure to thrive | Not evaluated | NM ^a^ | NM ^a^ | Fondaneche et al. (1998) (14) |
| 4 | NM ^a^ | Multiple bacterial infections | Homozygous p. F961S | NM ^a^ | NM ^a^ | Quan et al. (1999) (15) |
| 5 | Turkish | NM ^a^ | Homozygous c. 84bp del 3265-3348 | NM ^a^ | NM ^a^ | Peijnenburg et al. (2000) (16) |
| 6 | Greek | Pneumonia, septicemia, recurrent respiratory infections, recurrent gastroenteritis | Homozygous c.T1524C (p. L469P) | Supportive care | survived | Wiszniewski et al. (2001) (17) |
| 7 | NM ^a^ | Chronic diarrhoea, respiratory infections | No mutations in neither coding nor non-coding regions | HSCT | Died of GVHD or infection | Dziembowska et al.(2002) (18) |
| 8 | NM ^a^ | Chronic diarrhoea, respiratory infections | Allele 1: del3003-3084 Allele 2: CATdel3193-5 | HSCT | Died of CMV infection | Dziembowska et al.(2002) (18) |
| 9 | NM ^a^ | Chronic diarrhoea, respiratory infections | Heterozygous c.G2178A (p. Trp to X) Paternal allele: only silent mutations | HSCT | Died of CMV infection | Dziembowska et al.(2002) (18) |
| 10 | Austrian | Acute viral bronchiolitis and pneumonia | Homozygous p. Glu381X | NM ^a^ | NM ^a^ | Schmetterer et al. (2010) (19) |
| 11 | Mexican-Iranian | Disseminated M. avium complex, mild intellectual disability | Homozygous c.3317 + 1 G> A | Supportive care | NM ^a^ | Dimitrova et al. (2014) (20) |

^a^ Not mentioned
